# A New Approach towards Minimizing the Risk of Misdosing Warfarin Initiation Doses

**DOI:** 10.1155/2018/5340845

**Published:** 2018-05-13

**Authors:** Ashkan Sharabiani, Edith A. Nutescu, William L. Galanter, Houshang Darabi

**Affiliations:** ^1^Department of Mechanical and Industrial Engineering, University of Illinois at Chicago, Chicago, IL, USA; ^2^Department of Pharmacy Systems Outcomes and Policy and Center for Pharmacoepidemiology and Pharmacoeconomic Research, University of Illinois at Chicago, Chicago, IL, USA; ^3^Department of Medicine, University of Illinois at Chicago, Chicago, IL, USA

## Abstract

It is a challenge to be able to prescribe the optimal initial dose of warfarin. There have been many studies focused on an efficient strategy to determine the optimal initial dose. Numerous clinical, genetic, and environmental factors affect the warfarin dose response. In practice, it is common that the initial warfarin dose is substantially different from the stable maintenance dose, which may increase the risk of bleeding or thrombosis prior to achieving the stable maintenance dose. In order to minimize the risk of misdosing, despite popular warfarin dose prediction models in the literature which create dose predictions solely based on patients' attributes, we have taken physicians' opinions towards the initial dose into consideration. The initial doses selected by clinicians, along with other standard clinical factors, are used to determine an estimate of the difference between the initial dose and estimated maintenance dose using shrinkage methods. The selected shrinkage method was LASSO (Least Absolute Shrinkage and Selection Operator). The estimated maintenance dose was more accurate than the original initial dose, the dose predicted by a linear model without involving the clinicians initial dose, and the values predicted by the most commonly used model in the literature, the Gage clinical model.

## 1. Introduction

Warfarin is a commonly used oral anticoagulant drug with over 30 million prescriptions written annually in the United States [1]. This drug is difficult to manage because of its narrow therapeutic index and wide interpatient variability in dose response. Warfarin is the leading cause of drug-related hospitalizations among adults in the United States [2]. There are numerous factors affecting the activity of warfarin. They vary from each individual patient's characteristics, such as height, weight, age, and race, to the patient's medical history, diet, genotype, such as VKORC1 and CYP2C9, and their concurrent medications. Since various factors impact warfarin's dose response, numerous mathematical prediction models have been proposed to assist clinicians in finding the optimal initial dose [3–18]. The models that only contain clinical variables are known as clinical models (CL); models which also contain the patients' genotype are known as pharmacogenetic models (PKG).

Gage et al. [3] proposed two linear multiple regression models (CL & PKG models) in 2008. The clinical factors that were incorporated in both models are body surface area, target INR (International Normalized Ratio), smoking status, age, race, amiodarone use, and indication of VTE (Venous Thromboembolism). The IWPC (International Warfarin Pharmacogenetics Consortium) research group [4] also proposed two linear regression models. The models' performances were satisfactory for the patients who required doses less than or equal to 21 mg/wk or more than or equal to 49 mg/wk. According to the “Clinical Pharmacogenetics Implementation Consortium Guidelines for CYP2C9 and VKORC1 Genotypes and Warfarin Dosing,” the models proposed by Gage and IWPC are the most recommended models for predicting warfarin initiation doses [5]. Additionally, Grossi et al. [6] designed a novel model in Artificial Neural Networks (ANN) framework. After collecting the data of 377 patients, they derived the model and chose their variables using the TWIST system [7], which is designed in order to select the most relevant features for performing classification or prediction. They compared their model with IWPC and Gage's models, along with another model proposed by Zambon et al. [8] based on Mean Absolute Error (MAE) and model's fitness (*R*^2^), which proved their model's outperformance.

In 2015, Sharabiani et al. [9, 10] developed a new methodology towards estimating the initial warfarin dose. The proposed methodology estimates the initial dose for warfarin in two stages. In the first stage, using relevance vector machines, the patients are classified into two classes: patients requiring high doses (>30 mg/wk) and patients who require low doses (≤30 mg/wk). In the second stage, the dose for each class is predicted using two clinical regression models, which are trained for each class. Their proposed model was examined against Gage, IWPC clinical models, the fixed-dose approach (35 mg/wk), and the model proposed by Sharabiani et al. [10, 18] for African American patients, and it outperformed all of them in terms of prediction accuracy.

Several prediction models have also been proposed which target patients of specific ethnicities. The general proposed models are much more accurate when they are applied to Caucasian and Asian patients and less accurate in African American patients [11–13].

Using machine learning techniques, Cosgun et al. [14] proposed three PKG prediction models for African American patients. The models that were investigated were Boosted Regression Tree (BRT), Random Forest Regression (RFR), and Support Vector Regression (SVR). They compared their models to the models proposed by Schelleman et al. [15, 16] and Limdi et al. [13, 17] and reported their outperformance based on *R*^2^. Sharabiani et al. [10, 18] suggested a new clinical model for African American patients in 2013; the proposed model outperformed IWPC and Gage models in terms of prediction accuracy MAE and Root Mean Squared Error (RMSE). Hernandez et al. [19] also proposed a PKG model for African American patients. They used the data of 349 patients for training the model and 149 patients for validating it. They proved that their proposed model outperformed the PKG and CL models proposed by IWPC [4].

Clinicians are now faced with several alternative dosing approaches in order to determine the initiation dose of warfarin in clinical practice. They can use the loading dose method and the dose prediction models that are proposed in the literature or rely solely on their clinical knowledge and expertise.

The objective of this paper is to propose a method to minimize warfarin misdosing when the prescription of the initial dose is guided by clinical judgment alone.

The risk of misdosing is defined as a clinically significant percentage difference between the initial dose and the therapeutic dose. The therapeutic dose is defined as the dose leading to two consecutive INRs in the therapeutic INR range for at least 14 days apart. By prescribing an initial dose close to the therapeutic dose, the time to reach the target INR decreases and the risk of anticoagulant related complications such as bleeding and thrombosis can be reduced.

Since the definition of a “clinically significant percentage difference” is subject to individual interpretation, we have examined our procedure based on different scenarios. The proposed model estimates the amount of percentage error which can be either positive (in case of overdose) or negative (in case of underdose). Once the amount of percentage error is estimated, the optimal initial dose can be determined by revising the prescribed initial dose accordingly. If the estimated percentage error is not considered significant, the prescribed dose will be used unaltered. It is shown that, by using the proposed method, the risk of misdosing decreases significantly.

## 2. Materials and Methods

The dataset, which was used for this project, contains the data of 150 warfarin-treated patients in the University of Illinois Hospital & Health Sciences System (UI-Health) who had reached the therapeutic warfarin dose in their course of treatment.

At the University of Illinois Hospital (UIH), the ordering clinicians select the initial dose of warfarin. If the resulting dose from the Gage clinical model [3] which is calculated using data in the electronic medical record (EMR) and the dose selected by the clinician are more than 20% different from the calculated dose, a warning is shown to the ordering clinician which includes the suggested dose. The ordering clinician is free to accept or reject this dose. If the ordering clinician chooses to order warfarin pharmacogenetic testing, a pharmacogenetics service pharmacist will assist with future doses of warfarin. Otherwise, the clinical team will manage the dosing of warfarin.

**Figure 1:**
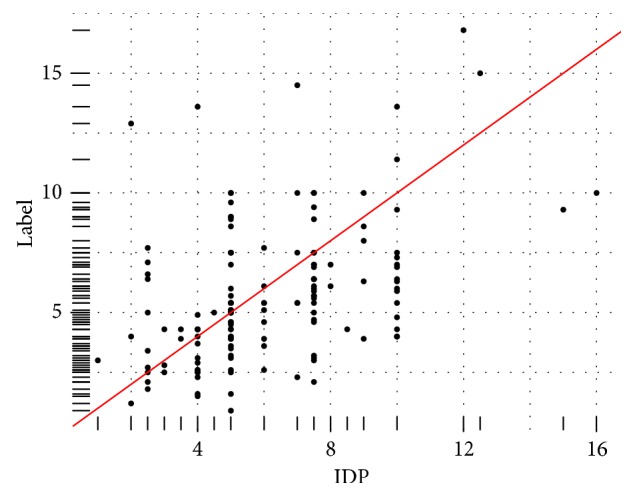
Distribution of the Initial Dose Prescribed by the Physician (IDP) Versus the Therapeutic Dose (Label).

Numerous patient variables were recorded. The variables in the dataset and their frequencies are presented in Tables 1 and 2. As a small minority of our patient population, our model may not be appropriate in Asians. In Figure 1, the correlation between Initial Dose Prescribed by the Physician (IDP) and therapeutic dose is presented. The red line, in Figures 1, 3, and 4, indicates the ideal dosing scenario for each patient which is a case of achieving a complete correlation between the two variables. It provides a visual aid as to how distant the points are in the space from the complete correlation between the variables.

**Figure 2:**
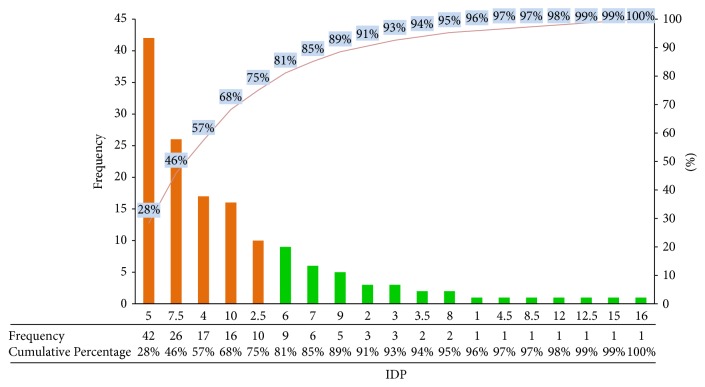
Pareto Chart for identifying the popular Initial Dose Prescribed by the Physician (IDP). 75% of the patients receive the popular doses (5, 7.5, 4, 10, 2.5 mg).

It is evident that most physicians tend to prescribe doses at popular discrete dose values. A Pareto chart measures this tendency as shown in in Figure 2.

**Figure 3:**
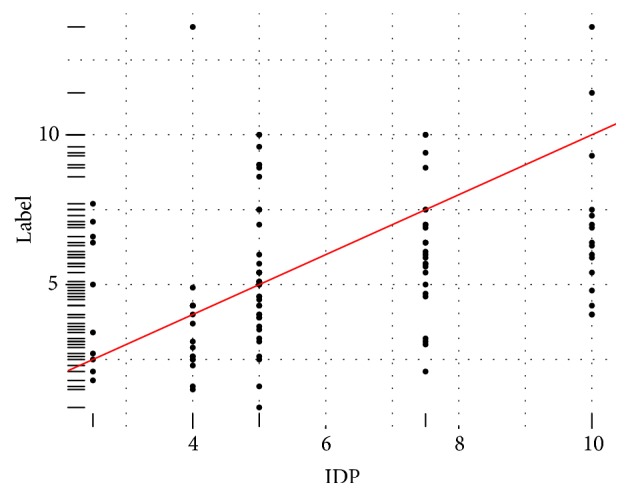
Distribution of the Initial Dose Prescribed by the Physician (IDP) versus the therapeutic dose (Label) for the popular doses against the ideal dosing setting.

**Figure 4:**
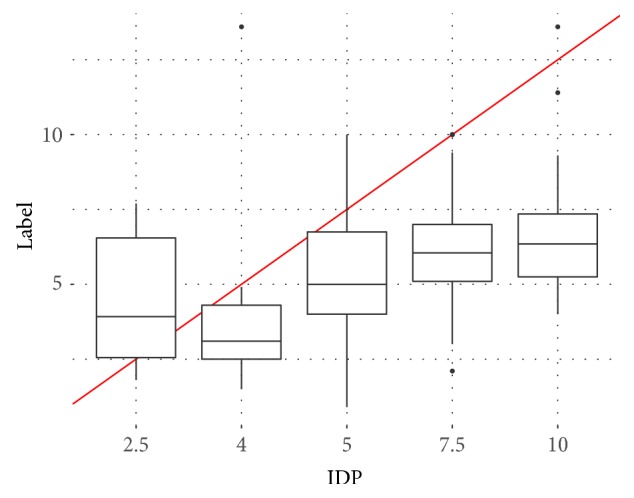
Comparing the distribution of therapeutic dose for popular Initial Dose Prescribed by the Physicians (IDP) using boxplots.

As it is presented in Figure 2, 75% of patients in the dataset received dose values of 2.5, 4, 5, 7.5, and 10 mg/day (bars colored in orange). We focused on the patients who have received those common doses. This was done to minimize the effect on rare unusual doses on our model and to increase the robustness of the model for the more common doses. This was necessary due to the relatively small size of the dataset. The distribution of the therapeutic dose at each level of the IDP is presented in Figure 3. Additionally, in Figure 4, a boxplot for each level is created.

**Figure 5:**
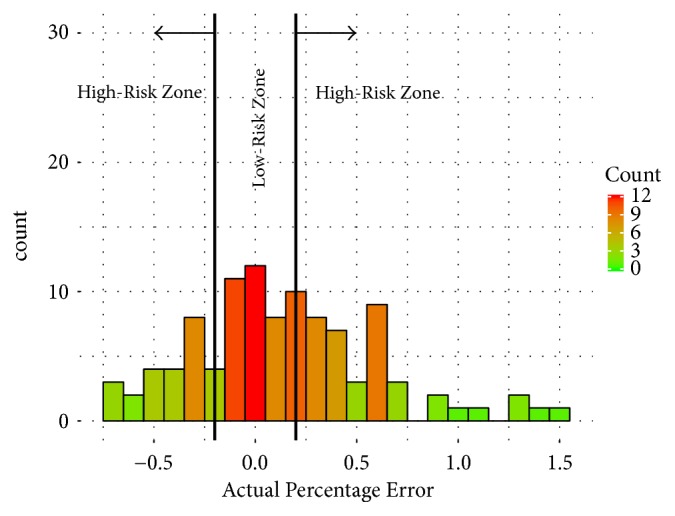
Defining the high-risk and low-risk dosing zones with the respect to the amount of generated percentage error by the Initial Dose Prescribed by the Physicians (IDP).

Using the initial dose which was prescribed by the clinicians and the value of the therapeutic dose, the amount of percentage error is calculated. The frequency of patients with differing percentage error is presented in Figure 4. By a subjective definition of a clinically significant percentage difference, the patients who are at high risk/low risk of misdosing can be identified. Taking Figure 5 as an example, it is assumed that 20% difference is a significant difference and it is shown by dark vertical lines. The bars in Figure 5 are color-coded based on the intensity of their corresponding volume.

**Figure 6:**
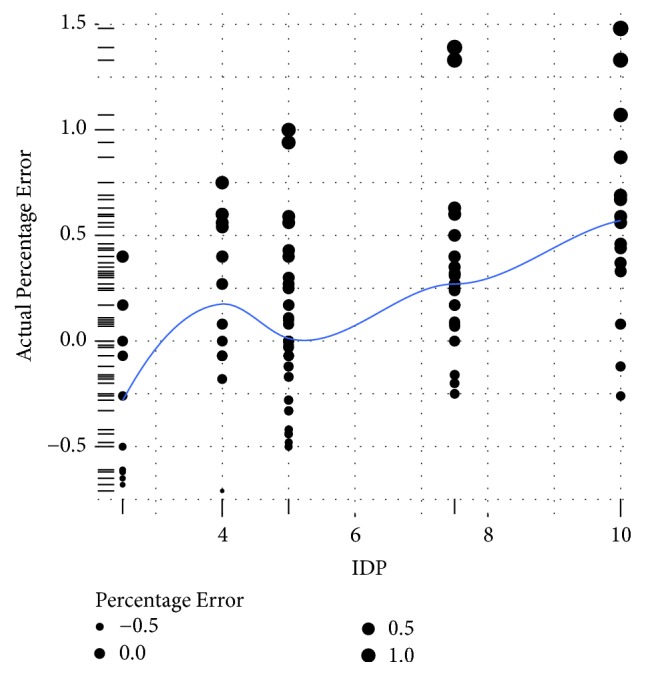
Distribution of percentage error at each level of popular Initial Doses Prescribed by the Physicians (IDP) (the sizes of the points are proportional with the amount of error generated at each dosing level).

Another point of interest is to identify the ranges of prescribed initial dose where higher values of percentage error occur. In Figure 3, the relationship between the initial dose and the percentage error is presented. Additionally, using a polynomial local regression, the fitted curve describing their relationship along with its prediction confidence interval is presented in Figure 6. The size of each point in Figure 6 is proportional to the amount of percentage error. It is evident from Figure 6 that the frequency of higher values of percentage error tends to increase at larger values of initial dose.

**Figure 7:**
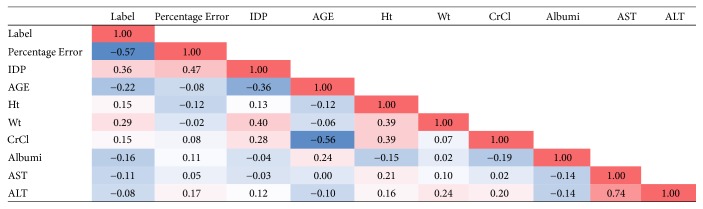
The Pearson correlation matrix. The values are color-coded to identify the highly correlated variables.

**Figure 8:**
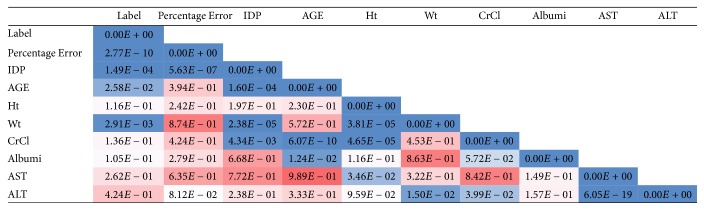
Corresponding *p* values to the Pearson correlation matrix in Figure 7.

Our goal is to develop a prediction model which assigns potential risk of misdosing to any prescribed initial dose. Therefore, in order to identify the linear dependency among the variables, a Pearson correlation matrix was created in Figure 7 and the corresponding *p* values are presented in Figure 8. The values in Figures 7 and 8 are color-coded to facilitate the process of comparing the relative magnitude of numbers in the figure with the dark red being the highest value and the dark blue being the lowest value. In order to avoid collinearity in modeling, variables that had a correlation more than or equal to 85% were defined as highly correlated; only one of them was entered in the modeling phase. The data points which had missing values for their therapeutic dose were eliminated from the dataset. The missing values for other variables were imputed using KNN (*K* = 5) method since 81% of the data points in the dataset were complete. The choice of *K* in the KNN resulted from the cross-validation process. There existed a significant number of variables compared to the number of data points in the dataset, so we needed to select the best subset of variables. Using shrinkage methods, the process of variable selection and developing a prediction model took place simultaneously. Accordingly, the categorical variables in the dataset were transformed into multiple binary dummy variables with one level kept out as the reference (baseline). In the data preprocessing phase, entering the two-level categorical variables with highly imbalanced ratio of levels (when the volume of one level is less than 10% of the entire values of the variable) was avoided. After dividing the data randomly to derivation and validation cohorts (60%/40%) the optimal prediction model was developed using LASSO (Least Absolute Shrinkage and Selection Operator) and the entire analysis was implemented in R 3.0.2. A brief overview of the shrinkage regression models is presented below.

### 2.1. Shrinkage Regression

An alternative approach to least square method, and ridge regression, towards estimating a linear model's coefficients, is LASSO (Least Absolute Shrinkage and Selection Operator) [18]. The objective in LASSO is to minimize the residual sum of square subjects through the summation of the absolute values of coefficients which are less than a constant.

(1) argmin ∑i=1Nyi−β0−∑jβjxij2Subject  to ∑jβj≤λ.

One of the most important characteristics associated with LASSO is that it enforces some coefficients to be exactly equal to zero and, hence, it results in a sparse model. However, by choosing a significantly small *λ*, this property will be nullified (and LASSO regression will be the regular least square model). Therefore, an appropriate choice of *λ* is quite critical. Because of this important attribute, the variable selection and modeling phases take place simultaneously. This idea can be considered as a major improvement over ridge regression where some coefficients will tend to zero but not exactly zero (see (2)).

(2) argmin ∑i=1Nyi−β0−∑jβjxij2Subject  to ∑jβj2≤λ.

Another major advantage of LASSO is its interpretability. As opposed to some more complex nonlinear models such as neural networks, LASSO will result in an interpretable model which is very important especially in clinical studies. For a detailed study on LASSO see Tibshirani's [19] original paper.

## 3. Results

**Figure 9:**
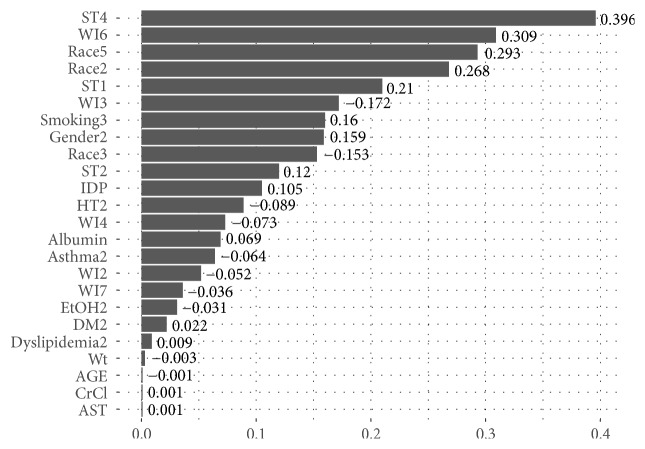
Model coefficients resulting from LASSO with involving IDP (the numbers attached to the variable names are the codes created after converting the variables into dummy variables. The codes are defined in Table 1.).

The optimal value of *λ* was selected by performing the *k*-fold cross-validation (*k* = 10). The resulting prediction model's coefficients are presented in Figure 9.

After developing the prediction model using the training set, its performance was evaluated on the testing set. Therefore, for every data point in the testing set the amount of percentage error was estimated. By defining a given threshold for determination of the significant percentage error, it can be decided whether the IDP was acceptable or would have needed modification. Therefore, the threshold represents the user's choice in defining the level of significance in percentage difference which triggers that action for dose revision.

According to the estimated percentage error, the prescribed initial dose can be revised.

(3) Revised  Dose=1−Estimated  Percentage  Error100×IDP.

Therefore, the resulting revised initial dose values were compared against the original initial dose along with the Gage model in terms of RMSE.

The RMSE is used as the leading indicator of modeling performance and was selected since it is more appropriate to use (than Mean Absolute Error) when the error has a Gaussian distribution. According to the most cited dataset in the literature for warfarin dosing, IWPC dataset [20], the assumption of the errors having the Gaussian distribution in a larger setting was proven and therefore most prediction models in this context in the literature are compared based on the RMSE.

**Figure 10:**
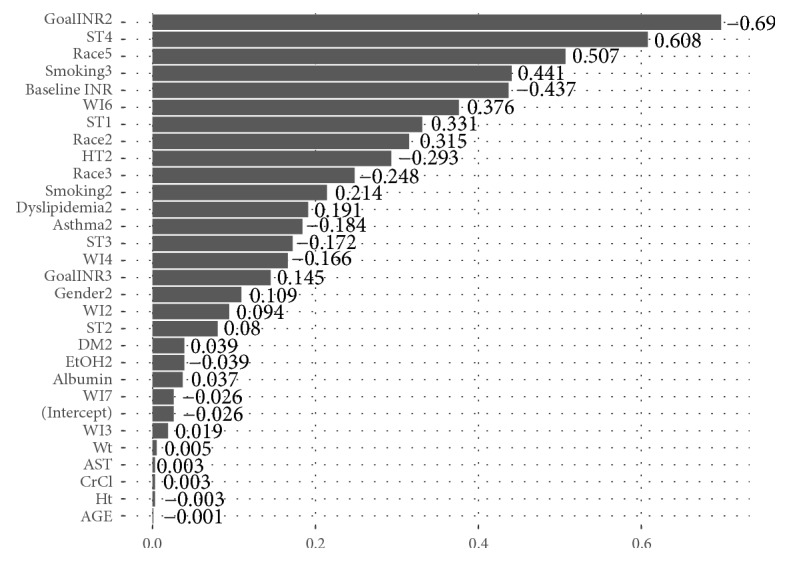
Model coefficients resulting from LASSO without involving IDP (the numbers attached to the variable names are the codes created after converting the variables into dummy variables. The codes are defined in Table 1.).

Additionally, in order to examine the impact of involving IDP in the modeling process, a new prediction model was developed with IDP being eliminated from the model. The developed model coefficients are presented in Figure 10.

Based on the results presented in Table 3, the estimated initial doses will result in more accurate estimations than the original dose values (RMSE = 2.38), the prediction values made by Gage model (RMSE = 2.05), and the linear model without using the initial dose in modeling (RMSE = 2.68).

## 4. Discussion

The proposed methodology has been developed and tested on the data of patients and physicians at the UIH, a tertiary urban hospital with an ethnically diverse patient population. The dataset is relatively small, but the 60% training set was able to develop a model which had better predictive power than traditional models. The methodology is novel for two reasons. First, this is the only model in the literature which uses information from clinicians, their first dose, to help estimate the best dose. Secondly, this model used a LASSO methodology to help deal with a less-than-ideal number of variables versus data points.

The goal of this project was to provide evidence of the feasibility of this approach. We have shown that there is some information content in the first dose ordered, as inclusion provides a better fit in our own model without the initial dose, as well as a better fit than traditional models. The reason that this occurred was not studied, but it suggests the previous models do not contain some variables or factors which ordering clinicians may be considering when dosing. Since the main focus of this study is to propose a new template for dosing by involving the suggested dose by the physician into the modeling process, for future deployment of such template, it is suggested to explore the performance of other predictive models after fully evaluating the model (power tests, diagnostic tests, etc.) as well. Additionally, on larger datasets, we suggest adjusting the *K*-fold cross-validation approach with lower number for *K* in order to avoid overfitting the model.

The final model produced by the LASSO procedure does not include some elements of the traditional clinical Gage equation, the use of the medications amiodarone, sulfamethoxazole, and azole antifungal agents. This may be due to the small population size and infrequency of use of these medications. Using this methodology on a larger dataset may or may not have the same finding. Our model includes the IDP which is not used in the Gage equation, and although it is less of a factor (see Figure 9) it does include the presence of diabetes mellitus. The Gage equation uses the presence or absence of liver disease, while our equation includes the lab values of AST and albumin, which can be considered a proxy for liver disease.

This type of analysis could be used for an active clinical decision support system once validated more thoroughly. All prior dosing data at a given institution which includes a proven maintenance dose can be used to develop the model. Once developed, a clinician's first dose, along with the noted patient variables would be used to determine a percentage error estimate. If this was less than some institutionally agreed upon threshold, no advice would be given to the clinician regarding their dose selection. If the initial dose was greater than the threshold away from the estimate, a dosing reminder or a new order could be introduced to the ordering clinician. This would only interrupt clinicians when there was a high likelihood of error.

The model is based on patient specific data from a given institution as well as the IDP for that institution. Because of this, it will need to be derived for each institution and as changes in initial dosing (IDP) occur, the model would likely change as the IDP does play a role in the predicted dose. If clinicians begin to dose purely on the model itself, it is likely that the added predictive power of adding the IDP will be lost as the variance between a predicted dose and the actual dose clearly contains useful information. It is however unlikely that this will occur at most hospitals soon as clinicians often disregard suggested dosing. As variations occur in physician compliance with initial dosing recommendations, patient mix, and changes in clinical practice, the model will need to be continuously adjusted. A reliance on prior clinical practice and prior patient mix will not likely produce the most accurate predictions.

The major limitation of this work is the relatively small dataset. The degree of fit and the novel use of the clinicians' first dose are intriguing and suggest larger studies to better validate the method. As this method is presently designed to help with the first dose, it can be used in conjunction with other models for subsequent decision support, with or without pharmacogenomics testing. It should work with any ethnic mixture of patients as the machine learning models are based on the patients seen at the particular institution, not a cohort from a published study which is likely different than the patients seen at an institution.

## 5. Conclusions

In this paper, an intelligent clinical decision support system for prescribing the initial dose of warfarin is presented. The maintenance dose of warfarin is estimated using shrinkage methods and including the actual initial ordered dose. This estimate was more accurate than the original dose given and the values predicted by the Gage clinical model. This approach is promising and warrants further study that may produce a functional clinical decision support system to assist with initial dosing of warfarin. The major limitation of this analysis is the small sample size used in its derivation. This limits the generalizability of our findings; however, the method is novel and should be tested in larger datasets. The proposed methodology serves as a modeling template for other healthcare institutions. Therefore, based on each institution's attributes (local patient's attributes/physicians' preferred dosing methods), customized models can be derived, which function more efficiently than generic models in the literature.

## Figures and Tables

**Table 1:** Categorical variables in the dataset.

Variable name	Values	Code	Frequency	Percentage
Race	African American	1	79	53%
	Hispanic	2	34	23%
	White	3	18	12%
	Asian	4	4	3%
	Others^*∗*^	5	15	10%

Gender	Male	1	67	45%
	Female	2	83	55%

Liver disease	Yes	1	3	2%
	No	2	125	83%
	Missing	NA	22	15%

Warfarin Indication (WI)	A.fib	1	25	17%
	DVT	2	53	35%
	PE	3	34	23%
	TKA/THA	4	13	9%
	MVR	5	1	1%
	CVA	6	4	3%
	Others	7	20	13%

Goal INR	2-3	1	136	91%
	2.5–3.5	2	3	2%
	1.8–2.5	3	11	7%

Amiodarone	Yes	1	5	3%
	No	2	144	96%
	Missing	NA	1	1%

Bactrim	Yes	1	1	1%
	No	2	148	99%
	Missing	NA	1	1%

Azole	Yes	1	1	1%
	No	2	148	99%
	Missing	NA	1	1%

Which statin? (ST)	None	0	93	62%
	Simva	1	14	9%
	Atorva	2	23	15%
	Prava	3	7	5%
	Lova	4	8	5%
	Rosuva	5	4	3%
	Missing	NA	1	1%

Dialysis	Yes	1	8	5%
	No	2	142	95%

Rheumatoid arthritis	Yes	1	1	1%
	No	2	149	99%

Collagen vascular disease	Yes	1	2	1%
	No	2	148	99%

Deep Vein Thrombosis (DVT)	Yes	1	10	7%
	No	2	140	93%

Smoking	Current smoker	1	13	9%
	Never smoker	2	107	71%
	Ex-smoker	3	30	20%

EtOH	Yes	1	24	16%
	No	2	119	79%
	Missing	NA	7	5%

Illicit	Yes	1	6	4%
	No	2	144	96%

Hypertension	Yes	1	86	57%
	No	2	64	43%

Angina	Yes	1	1	1%
	No	2	149	99%

Myocardial Infarction	Yes	1	3	2%
	No	2	147	98%

Percutaneous Coronary Intervention (PCI)	Yes	1	6	4%
	No	2	144	96%

Coronary Artery Bypass Graft (CABG)	Yes	1	5	3%
	No	2	145	97%

Atrial Fibrillation or Flutter	Yes	1	11	7%
	No	2	139	93%

Diabetes Mellitus (DM)	Yes	1	48	32%
	No	2	102	68%

Stroke	Yes	1	11	7%
	No	2	139	93%

Chronic Renal Insufficiency	Yes	1	15	10%
	No	2	135	90%

Chronic Obstructive Pulmonary Disease (COPD)	Yes	1	7	5%
	No	2	143	95%

Asthma	Yes	1	18	12%
	No	2	132	88%

Valvular heart disease	Yes	1	1	1%
	No	2	149	99%

Sickle cell	Yes	1	3	2%
	No	2	147	98%

Cancer history	Yes	1	12	8%
	No	2	138	92%

Pulmonary Embolism (PE)	Yes	1	5	3%
	No	2	144	96%
	Missing	NA	1	1%

Dyslipidemia	Yes	1	53	35%
	No	2	97	64%

Heart Failure (HF)	Yes	1	15	10%
	No	2	135	90%

Peripheral Vascular Disease (PVD)	Yes	1	7	4%
	No	2	143	95%

^*∗*^These patients have predominantly unknown race.

**Table 2:** Continuous variables in the data set.

Continuous Variables	Unit	Number of missing instances	Mean	Median	Standard deviation	Min	Max
Therapeutic dose (Label)	mg/day	0	5.68	2.87	5.1	0.9	16.8
Initial Dose Prescribed By the Physician (IDP)	mg/day	2	6.12	2.59	5	1	16
Percentage error		2	0.26	0.7	0.12	−0.84	4.83
Age	Year	0	54.29	17.82	57	18	91
Height (Ht )	cm	0	168.28	10.35	169	142.2	195
Weight (Wt)	kg	0	89.9	31.12	83	40	220
Creatinine Clearance (CrCl)	ml/min	2	64.79	36.32	63.65	3.6	146.5
Albumin	g/dl	17	3.12	0.65	3.2	1.4	4.3
Aspartate Aminotransferase (AST)	u/L	22	33.56	41.04	22	9	379
Alanine Aminotransferase (ALT)	u/L	22	25.88	24.85	19	5	199
Baseline INR		1	1.18	0.14	1.2	1	1.8

**Table 3:** Comparing the performance of the revised values of IDP with the original values of IDP, the Gage CL model, and the linear model without IDP. The percentage change in the table represents the percentage decrease in RMSE after revising the IDP.

Threshold	RMSE of the revised IDP	RMSE of the Original IDP (percent change)	RMSE of the Gage model (percent change)	RMSE of the linear model without involving IDP (percent change)
0.1	1.65	2.391 (31%)	2.063 (20%)	2.661 (38%)
0.15	1.76	2.378 (26%)	2.047 (14%)	2.667 (34%)
0.2	1.77	2.392 (26%)	2.051 (13.7%)	2.682 (34%)
0.25	1.9	2.375 (20%)	2.05 (7.3%)	2.68 (29.1%)
0.3	1.96	2.39 (18%)	2.05 (4.4%)	2.681 (26.9%)
0.35	1.96	2.39 (18%)	2.05 (4.4%)	2.681 (26.9%)
0.4	2.06	2.368 (13%)	2.05 (−0.5%)	2.679 (23.1%)
